# Effects of Irradiance, Pressure, and Temperature on the Photodegradation of Fast Pressure-Sensitive Paints

**DOI:** 10.3390/ma19173732

**Published:** 2026-09-02

**Authors:** Feng Gu, Mingkai Xue, Zichao Zhu, Yingzheng Liu, Di Peng

**Affiliations:** 1 Key Laboratory of Education Ministry for Power Machinery and Engineering, School of Mechanical Engineering, Shanghai Jiao Tong University, Shanghai 200240, China; 707996506@sjtu.edu.cn (F.G.); bill_oct14@sjtu.edu.cn (M.X.); fitzzc_98@sjtu.edu.cn (Z.Z.); yzliu@sjtu.edu.cn (Y.L.); 2 Gas Turbine Research Institute, Shanghai Jiao Tong University, Shanghai 200240, China

**Keywords:** photodegradation, polymer–ceramic PSP, mesoporous-particle PSP, irradiance

## Abstract

Photodegradation limits the accuracy of fast pressure-sensitive paints (PSPs), but its dependences on irradiance and operating conditions remain insufficiently researched. Here, the photodegradation kinetics of a polymer–ceramic PSP (PC-PSP) and a mesoporous-particle PSP (MP-PSP) were investigated under independently varied irradiance (50–122 mW/cm^2^), pressure (0.5–100 kPa), and temperature (20–100 °C). Normalized intensity histories acquired during 900 s of continuous irradiation were fitted with a bi-exponential model, whose composite degradation rate was correlated to the studied parameters using physical relations. PC-PSP exhibited greater absolute degradation and an approximately linear irradiance dependence (m = 1.28), whereas MP-PSP showed a nonlinear dependence (m = 2.13), consistent with a greater relative contribution from photooxidative processes. The degradation rate of PC-PSP decreased predominantly with increasing pressure because oxygen quenching reduced the excited-state luminophore population. MP-PSP exhibited a non-monotonic pressure dependence: decreasing pressure initially accelerated degradation by weakening oxygen quenching, whereas further pressure reduction below the half-saturation region suppressed oxygen-limited photooxidation. Increasing temperature generally accelerated degradation in both formulations; the fitted apparent activation energies were 12.99 kJ mol^−1^ for PC-PSP and 33.12 kJ mol^−1^ for MP-PSP, reflecticng the stronger overall temperature dependence of MP-PSP. These findings provide a basis for quantitatively evaluating PSP photostability and assessing photodegradation-induced measurement errors.

## 1. Introduction

Pressure-sensitive paint (PSP) is an optical technique for full-field surface-pressure measurement with high spatial resolution [1,2,3,4,5]. Its sensing mechanism is based on the oxygen quenching of luminophore emission. Under a fixed gas composition, the oxygen concentration in the binder varies with oxygen partial pressure and, consequently, with surface pressure. Pressure is generally determined from the luminescence-intensity ratio between reference and test conditions using a Stern–Volmer equation [1]. This intensity-based method requires stable and continuous excitation, which however, causes irreversible photodegradation and a progressive decrease in luminescence intensity [6,7,8]. When the reference and test images are acquired at different stages of degradation, the resulting intensity change is interpreted as a pressure variation, introducing a systematic measurement error.

Photodegradation is particularly critical for fast-responding PSPs. To increase the rate of oxygen permeation through the binder, these paints generally employ highly porous structures with large specific surface areas [9,10,11,12,13,14]. Compared with conventional PSPs, the fast PSPs usually have a low polymer content, resulting in high local luminophore concentrations and heterogeneous microenvironments which markedly increase the photodegradation rate [15]. Moreover, time-resolved measurements generally require intense excitation to maintain an adequate signal-to-noise ratio with short exposure times. In addition, the PSP’s luminescent molecule can also act as a photosensitizer: energy transfer from its long-lived triplet excited state to ground-state molecular oxygen produces singlet oxygen, whose emission has been directly observed in PSP polymer films [16,17]. The generated singlet oxygen is highly reactive and may promote photooxidation of both the luminophore and the surrounding polymer matrix, thereby contributing to irreversible photodegradation [16,18]. These factors collectively increase the susceptibility of fast PSPs to photodegradation.

Several approaches have been proposed to mitigate PSP photodegradation. Nunn et al. examined the influence of porphyrin structure on PSP performance [19], whereas Basu et al. related the intensity loss of pyrene-based PSP to photochemical changes in the luminophore [20]. Egami and Asai distinguished oxygen-independent photodecomposition from oxygen-dependent photooxidation and evaluated antioxidants for suppressing these processes [21]. Subsequent studies examined the effects of substrates, binders, luminophore structures, and formulation parameters on photostability [22,23,24]. Mochizuki et al. immobilized luminophores in mesoporous silica nanoparticles to reduce photochemical interactions in a dual-luminophore PSP [25], whereas Uchida et al. improved the photostability of a dual-luminophore system by adding antioxidants [26]. Peng et al. developed an MP-PSP in which luminophores were deposited within mesoporous silica particles and reported substantially improved photostability compared with conventional PC-PSP [14]. They attributed this improvement to the reduced interaction between the luminophore and polymer and to the more uniform luminophore distribution within the binder.

Despite these advances, existing studies do not provide a comprehensive and quantitative description of PSP photodegradation. Most studies characterize photostability using endpoint intensity loss or a decay rate obtained under a single excitation condition [14,20,21,22,23,24,25,26]. These metrics combine the effects of test-specific factors, including irradiance, excitation spectrum, and exposure duration, making results from different experiments difficult to compare or extrapolate to other operating conditions. Moreover, endpoint intensity loss and linear decay rates cannot represent the rapid initial decrease and subsequent slower decay commonly observed during continuous irradiation. The relationship between irradiance and degradation rate therefore remains insufficiently quantified.

In addition, the effects of pressure and temperature introduce further complexity. As optical sensing molecules, PSP luminophores exhibit pressure and temperature sensitivities because their emission is affected by oxygen quenching and thermal nonradiative transitions. Increasing pressure or temperature will reduce the luminescence intensity [1,6,7]. These immediate photophysical responses are reversible and have been extensively characterized [10,13,14,15]. On the other hand, pressure and temperature also influence the irreversible photodegradation process, which constitutes the focus of the present study. Strasser et al. have demonstrated that PSP photodegradation varies with pressure, providing evidence that oxygen is involved in the degradation process [27]. Nevertheless, oxygen has competing effects: it reduces the population of excited-state luminophores through oxygen quenching but can also participate in photooxidation. The relationship between oxygen partial pressure and the overall degradation rate may therefore be nonlinear. Temperature affects oxygen solubility and diffusion as well as the rates of photochemical reactions, thereby influencing oxygen availability and the overall photodegradation kinetics [6,7,28,29]. However, the influence of pressure and temperature on photodegradation has received limited attention.

In this study, the photodegradation characteristics of two representative fast PSPs, PC-PSP and MP-PSP, are investigated under independently controlled irradiance, pressure, and temperature. The temporal intensity decay is described using a bi-exponential function, from which a composite degradation-rate parameter is derived. The dependences of this parameter on irradiance, pressure, and temperature are analyzed separately. The results provide a quantitative basis for evaluating PSP photostability, and may guide the selection of excitation irradiance, exposure duration, and operating conditions for time-resolved PSP measurements. The present study aims to quantify the individual influence of each variable on photodegradation kinetics, which is a necessary and meaningful step toward a more comprehensive investigation of their coupling effects.

## 2. Photodegradation Model of PSP

Under continuous irradiation, a fraction of the excited luminophore molecules undergoes irreversible photochemical reactions and loses its luminescence capability. The photodegradation of PSP generally involves oxygen-independent photodecomposition and oxygen-dependent photooxidation [21]. At constant pressure and temperature, the intensity decay is characterized by the normalized luminescence intensity:

(1) $$\begin{array}{c} R\left(t\right)=\frac{I\left(t\right)}{I_{0}}, \end{array}$$

where $I_{0}$ and $I\left(t\right)$ are the luminescence intensities at the beginning of irradiation and at irradiation time t, respectively. Normalization reduces differences in the initial intensity caused by variations in paint thickness and luminophore loading.

For fast PSPs, the luminophores are distributed among polymer-rich regions, ceramic or particle surfaces, and polymer–particle interfacial transition regions. These sites differ in oxygen accessibility, local luminophore concentration, and interaction with the binder [15]. The measured decay is therefore represented by a bi-exponential function:

(2) $$\begin{array}{c} R\left(t\right)=f\exp\left(-k_{1}t\right)+\left(1-f\right)\exp\left(-k_{2}t\right), \end{array}$$

where $k_{1}$ and $k_{2}$ are the fast and slow degradation-rate constants, respectively, and f is the initial contribution of the fast-decaying component [18,30]. The two components provide an effective description of the heterogeneous degradation kinetics and should not be assigned to two specific photochemical reactions or two discrete luminophore environments. The luminophore microenvironment, oxygen accessibility, self-quenching, and interactions with the polymer matrix are complex and strongly coupled, making the physical meanings of f, $k_{1}$, and $k_{2}$ difficult to interpret individually. Therefore, to provide a representative comparison of the overall degradation kinetics under different conditions, a composite degradation-rate constant is defined from the initial slope of Equation (2):

(3) $$\begin{array}{c} K_{D}=-{\left.\frac{dR\left(t\right)}{dt}\right|}_{t=0}=fk_{1}+\left(1-f\right)k_{2}. \end{array}$$

This parameter incorporates the contributions of both decay components and avoids using either $k_{1}$ or $k_{2}$ alone to characterize the overall photostability. The accumulated intensity loss after an irradiation period $t_{e}$ is separately defined as

(4) $$\begin{array}{c} D\left(t_{e}\right)=1-R\left(t_{e}\right), \end{array}$$

where D represents the final photodegradation ratio. Thus, $K_{D}$ characterizes the degradation kinetics, whereas D quantifies the resulting signal loss over a prescribed irradiation period.

Because photodegradation is initiated by photon absorption, its rate is expected to depend on the irradiation intensity. At fixed pressure and temperature, the irradiation-intensity dependence of $K_{D}$ is empirically represented by a power-law relation, following the generalized reciprocity approach commonly used in photodegradation studies [31,32]:

(5) $$\begin{array}{c} K_{D}\left(E_{irr}\right)=K_{D,ref}{\left(\frac{E_{irr}}{E_{irr,ref}}\right)}^{m}, \end{array}$$

where $E_{irr}$ is the irradiation intensity, $E_{irr,ref}$ is the reference irradiation intensity, $K_{D,ref}$ is the corresponding degradation-rate constant, and m is the irradiation-intensity exponent. A value of m=1 indicates that the degradation rate is proportional to the incident photon flux. A deviation from unity may result from excited-state quenching, reaction saturation, intermolecular interactions, or competing photochemical pathways. In PSPs, m>1 may occur when increasing irradiation promotes the accumulation of reactive intermediates (electronically excited oxygen molecules or polymer decomposition products) and accelerates luminophore photooxidation, whereas m<1 may arise when additional excitation is increasingly dissipated or degradation is limited by local oxygen concentration.

Pressure affects photodegradation by changing the oxygen concentration within the PSP. Oxygen then influences the degradation process through two competing mechanisms. Oxygen can participate in photooxidation, thereby increasing the degradation rate, but it can also reduce the excited-state luminophore population through oxygen quenching and consequently suppress irreversible photochemical reactions. To describe these effects separately, the oxygen-dependent photooxidation contribution is first represented using a half-saturation relation [33]:

(6) $$\theta_{O_{2}}=\frac{p_{O_{2}}}{p_{O_{2}}+p_{O_{2},1/2}},$$

where $\theta_{O_{2}}$ denotes the relative oxygen concentration for photooxidation. At low oxygen partial pressure, $\theta_{O_{2}}$ increases approximately linearly with $p_{O_{2}}$, whereas at high oxygen partial pressure it gradually approaches unity. Before normalization to the reference condition, the effective degradation rate can therefore be expressed as

(7) $$K_{D}\left(p_{O_{2}}\right)=\frac{K_{pd}+K_{ox}\theta_{O_{2}}}{1+K_{q}p_{O_{2}}}=\frac{K_{pd}+K_{ox}\frac{p_{O_{2}}}{p_{O_{2}}\;+\;p_{O_{2},1/2}}}{1+K_{q}p_{O_{2}}},$$

The numerator contains the oxygen-independent photodecomposition contribution, $K_{pd}$, and the oxygen-dependent photooxidation contribution, whose value is $K_{ox}\theta_{O_{2}}$, and $K_{q}$ is the effective oxygen-quenching coefficient. The denominator represents the reduction in the excited-state luminophore population caused by oxygen quenching [34,35]. Normalizing this expression by the degradation rate at the reference oxygen partial pressure and defining $\beta_{ox}=K_{ox}/K_{pd}$ gives

(8) $$K_{D}\left(p_{O_{2}}\right)=K_{D,ref}\frac{1+\beta_{ox}\frac{p_{O_{2}}}{p_{O_{2}}\;+\;p_{O_{2},1/2}}}{1+K_{q}p_{O_{2}}}\frac{1+K_{q}p_{O_{2},ref}}{1+\beta_{ox}\frac{p_{O_{2},ref}}{p_{O_{2},ref}\;+\;p_{O_{2},1/2}}},$$

Here, $p_{O_{2}}$ is the oxygen partial pressure, $p_{O_{2},ref}$ is the reference oxygen partial pressure, and $K_{D,ref}$ is the corresponding degradation-rate constant. The dimensionless parameter $\beta_{ox}$ represents the maximum photooxidation contribution relative to oxygen-independent photodecomposition. The parameter $p_{O_{2},1/2}$ is the effective oxygen half-saturation pressure. At $p_{O_{2}}=p_{O_{2},1/2}$, the oxygen-dependent photooxidation term reaches half of its limiting value. When $p_{O_{2}}\ll p_{O_{2},1/2}$, photooxidation is oxygen-limited and varies approximately linearly with oxygen partial pressure. When $p_{O_{2}}\gg p_{O_{2},1/2}$, the photooxidation contribution approaches saturation. Thus, $p_{O_{2},1/2}$ is an effective kinetic parameter describing oxygen concentration for photooxidation rather than the thermodynamic saturation concentration of oxygen in the binder. For experiments conducted in air,

(9) $$\begin{array}{c} p_{O_{2}}=x_{O_{2}}p, \end{array}$$

where $x_{O_{2}}$ is the oxygen mole fraction (0.21 in air) and p is the total pressure, and the reported pressures are absolute pressures. Equation (8) allows the degradation rate to increase, decrease, or approach a limiting value with increasing oxygen partial pressure, depending on the relative contributions of photooxidation and excited-state quenching.

Temperature affects the rates of photochemical reactions as well as oxygen transport and non-radiative deactivation within the binder. At fixed irradiation intensity and pressure, the temperature dependence of the composite degradation rate is described using an Arrhenius-type relation [36]:

(10) $$\begin{array}{c} K_{D}\left(T\right)=K_{D,ref}\exp\left[-\frac{E_{a}}{R_{g}}\left(\frac{1}{T}-\frac{1}{T_{ref}}\right)\right], \end{array}$$

where T and $T_{ref}$ are the absolute and reference temperatures in kelvin, $R_{g}$ is the universal gas constant, and $E_{a}$ is the apparent activation energy. The term “apparent” is used because the measured temperature dependence may include contributions from photodecomposition, photooxidation, oxygen transport, and the heterogeneous luminophore microenvironment rather than a single elementary reaction.

In summary, Equations (2)–(4) describe the temporal luminescence decay and define the composite degradation rate and accumulated intensity loss. Equation (5) describes the dependence of the degradation rate on irradiation intensity. Equations (6)–(9) describe the pressure dependence by considering oxygen-limited photooxidation, oxygen quenching, and the relationship between oxygen partial pressure and total pressure. Equation (10) describes the temperature dependence using an Arrhenius-type relation. Together, these equations describe irreversible luminescence decay under different irradiation, pressure and temperature conditions.

## 3. Materials and Experimental Methods

### 3.1. PSP Preparation

Platinum(II) meso-tetra(pentafluorophenyl)porphine (PtTFPP, Frontier Scientific, Logan, UT, USA) was used as the pressure-sensitive luminophore for both PSP formulations. The PC-PSP was prepared using commercial-grade rutile titania powder (nominal particle size: 300–400 nm; Macklin Biochemical, Shanghai, China), distilled water, and a commercial polyacrylate emulsion. The titania particles and distilled water were first mixed at a mass ratio of 1:1. Subsequently, 66.6 mg of the polymer emulsion was added to 1.0 g of the titania–water mixture, corresponding to a polymer ratio of 0.0666. This formulation was selected because it provided an appropriate compromise between pressure sensitivity, response time, and photostability [15]. The resulting slurry was stirred for 5 min and air-sprayed onto aluminum coupons. The samples were dried at 50 °C for 12 h. PtTFPP was then dissolved in dichloromethane at a concentration of 1 mg/mL, and 1 mL of the solution was air-sprayed onto each basecoat. The final coating thickness was approximately 50 μm.

The MP-PSP was prepared using the mix-and-spray method reported by Peng et al. [14], with poly(isobutyl methacrylate), hereafter abbreviated as poly(IBM), replacing the polystyrene used in the original formulation. Specifically, 40 mg of poly(IBM), 150 mg of mesoporous silica particles, and 1 mg of PtTFPP were added to 1 mL of dichloromethane. Tween 80 was then added at a concentration of 1–2% as a dispersant. The mixture was sonicated for 10 min to obtain a uniform slurry and subsequently air-sprayed onto aluminum coupons. The final coating thickness was approximately 60–70 μm.

All samples were stored in darkness after preparation to minimize photodegradation before testing. A total of 100 samples were prepared for each PSP formulation, and a fresh sample was used for each photodegradation experiment. The PC-PSP and MP-PSP samples were fabricated on identical substrates and tested using the same optical and environmental control system.

### 3.2. Experimental Setup and Procedure

**Figure 1:**
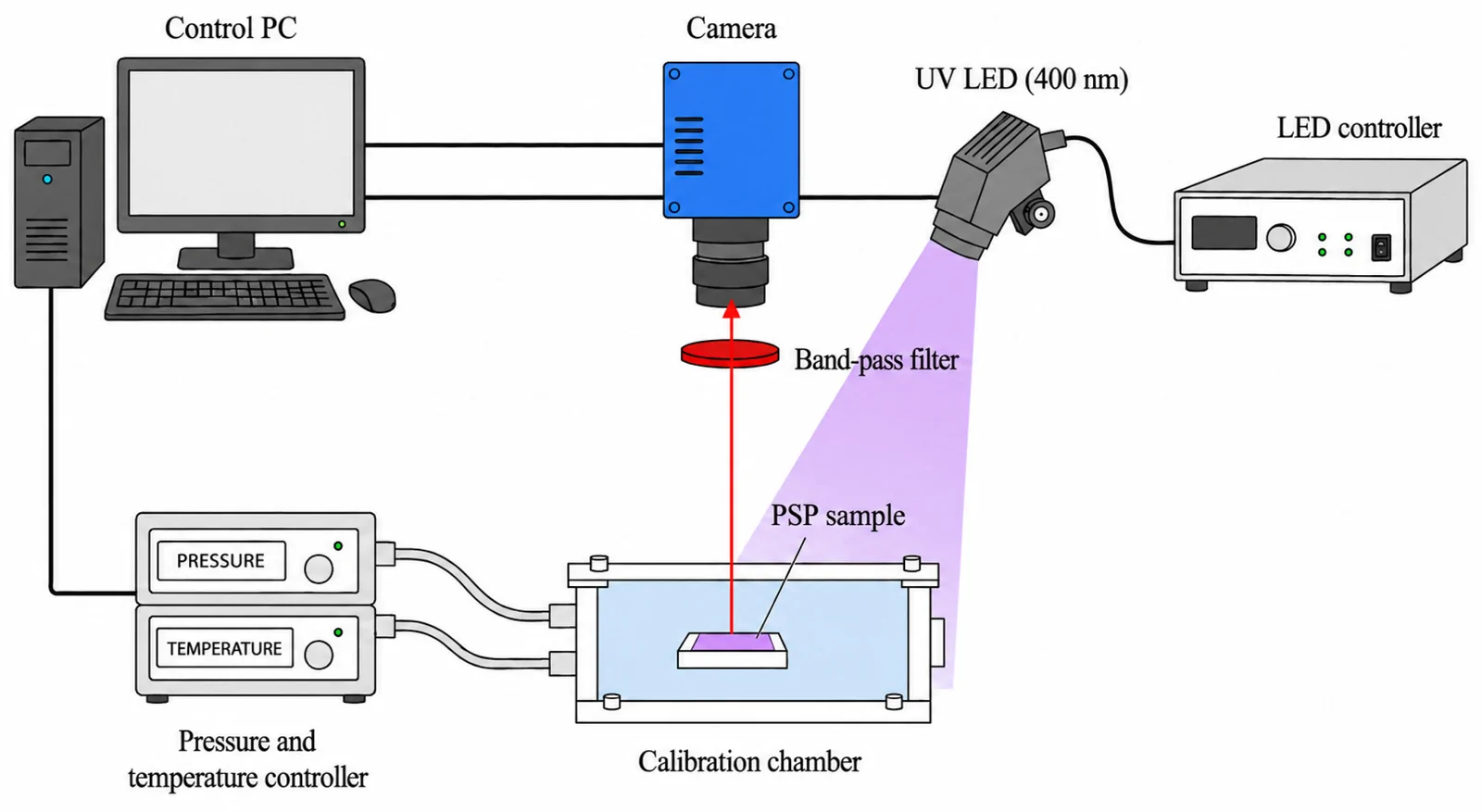
Schematic of the experimental setup for photodegradation measurements.

The photodegradation experiments were conducted in a temperature- and pressure-controlled chamber, as shown in Figure 1. The chamber was regulated using a pressure and temperature controller (ConST820, ConST, Beijing, China), providing pressure control from 0.5 to 200 kPa with an accuracy of 0.02 kPa and temperature control from −20 to 100 °C with an accuracy of 0.05 °C. The PSP samples were excited using an ultraviolet light-emitting diode (OP-EX50-400; Guangdong Academy of Sciences, Guangzhou, China) with a central wavelength of 400 nm and a nominal optical power of 50 W. The light-output stability exceeded 99.5%, corresponding to temporal fluctuations below 0.5%. Lumicnescence images were acquired using a CMOS camera (5 MP, 12 bit; HIKROBOT, Hefei, China) equipped with a lens (M3514-MP, 35 mm; Computar, Durham, NC, USA) and a band-pass filter (650 ± 25 nm; #84-798, Edmund Optics, Barrington, IL, USA) to reject the excitation light.

An in-house program was used to coordinate the pressure and temperature controller, excitation source, and camera. For each test, the target pressure and temperature were first sent to the controller. Data acquisition was initiated only after the deviations from the target pressure and temperature remained within 0.02 kPa and 0.05 °C, respectively, for at least 3 min. After the target condition had been reached and stabilized, the excitation source was switched on and maintained continuously for 15 min. The target pressure and temperature were continuously maintained throughout the subsequent 15 min irradiation and image-acquisition period, thereby excluding reversible intensity variations caused by temporal changes in pressure or temperature. The camera acquired one luminescence image every 1 s throughout the irradiation period. The aperture was fixed at f/2.0, while the exposure time was adjusted for each experimental condition to maintain the image intensity at the beginning of photodegradation at approximately 2000–3000 counts. At the end of the 15-min exposure, the excitation source and camera were switched off automatically. The same acquisition procedure was applied to all irradiation-intensity, pressure, and temperature conditions.

The effect of irradiance was investigated at pressures of 100 kPa and a temperature of 20 °C. The irradiation intensity was sequentially set to 50, 67, 83, 99, 115, and 122 mW/cm^2^, which are calibrated using an optical power meter. The effect of pressure was examined at 0.5, 5, 12.5, 25, 50, 75, and 100 kPa, while the irradiation intensity and temperature were maintained at 67 mW/cm^2^ and 20 °C, respectively. To further investigate how pressure affects the irradiance dependence of photodegradation, additional irradiance-series experiments were conducted at 10 and 1 kPa under the same experimental conditions as those used at 100 kPa. The temperature-dependent experiments were conducted at 20, 40, 60, 80, and 100 °C, with the pressure and irradiation intensity fixed at 100 kPa and 67 mW/cm^2^, respectively. Before the experiments, repeatability was evaluated for each formulation through three independent tests at 83 mW/cm^2^, 100 kPa, and 20 °C, with each test using a fresh sample. The relative uncertainties in the *K_D_* were 1.82% for PC-PSP and 4.66% for MP-PSP, respectively. Considering the cost associated with the full experimental matrix, a single experiment using one fresh sample was conducted for each test condition.

### 3.3. Data Processing

All images were processed using the same procedure. A dark image acquired with the excitation source switched off was first subtracted from each raw image. A fixed region of interest (30 × 30 pixel) was then selected for each sample, and its spatially averaged intensity was used for subsequent analysis.

For each photodegradation experiment, the intensity was normalized by that of the first recorded image according to Equation (1). The resulting decay curve was fitted using the bi-exponential model in Equation (2) through nonlinear least-squares regression. The fitting parameters were constrained by 0≤f≤1 and $k_{1}>k_{2}>0$. The composite degradation-rate constant and final photodegradation ratio were then calculated using Equations (3) and (4), respectively. The dependence of $K_{D}$ on irradiation intensity was fitted using Equation (5). The pressure- and temperature-dependent parameters were independently determined using Equations (8) and (10). In each case, only one experimental variable was varied, while the other conditions were maintained constant. The 95% confidence intervals of the fitted model parameters were estimated using residual bootstrap with 2000 resamples.

## 4. Results and Discussion

### 4.1. Effect of Irradiance

**Figure 2:**
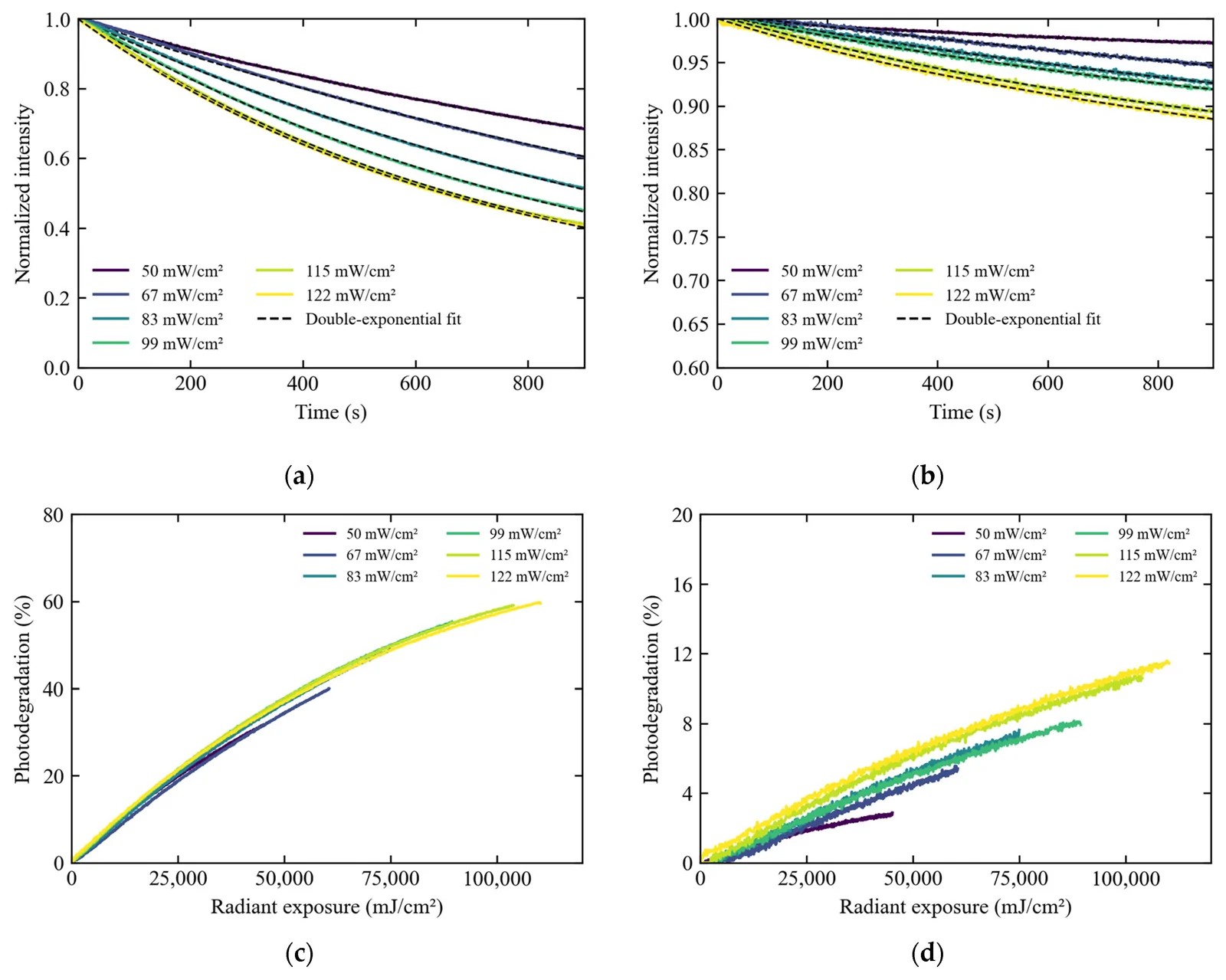
Normalized luminescence intensity histories and radiant-exposure-dependent photodegradation under different irradiation intensities. The upper row presents the normalized intensity histories of (**a**) PC-PSP and (**b**) MP-PSP; colored solid lines represent the experimental data, and black dashed lines represent the bi-exponential fits. The lower row presents the photodegradation ratio as a function of accumulated radiant exposure for (**c**) PC-PSP and (**d**) MP-PSP. All experiments were conducted at 100 kPa and 20 °C.

**Figure 3:**
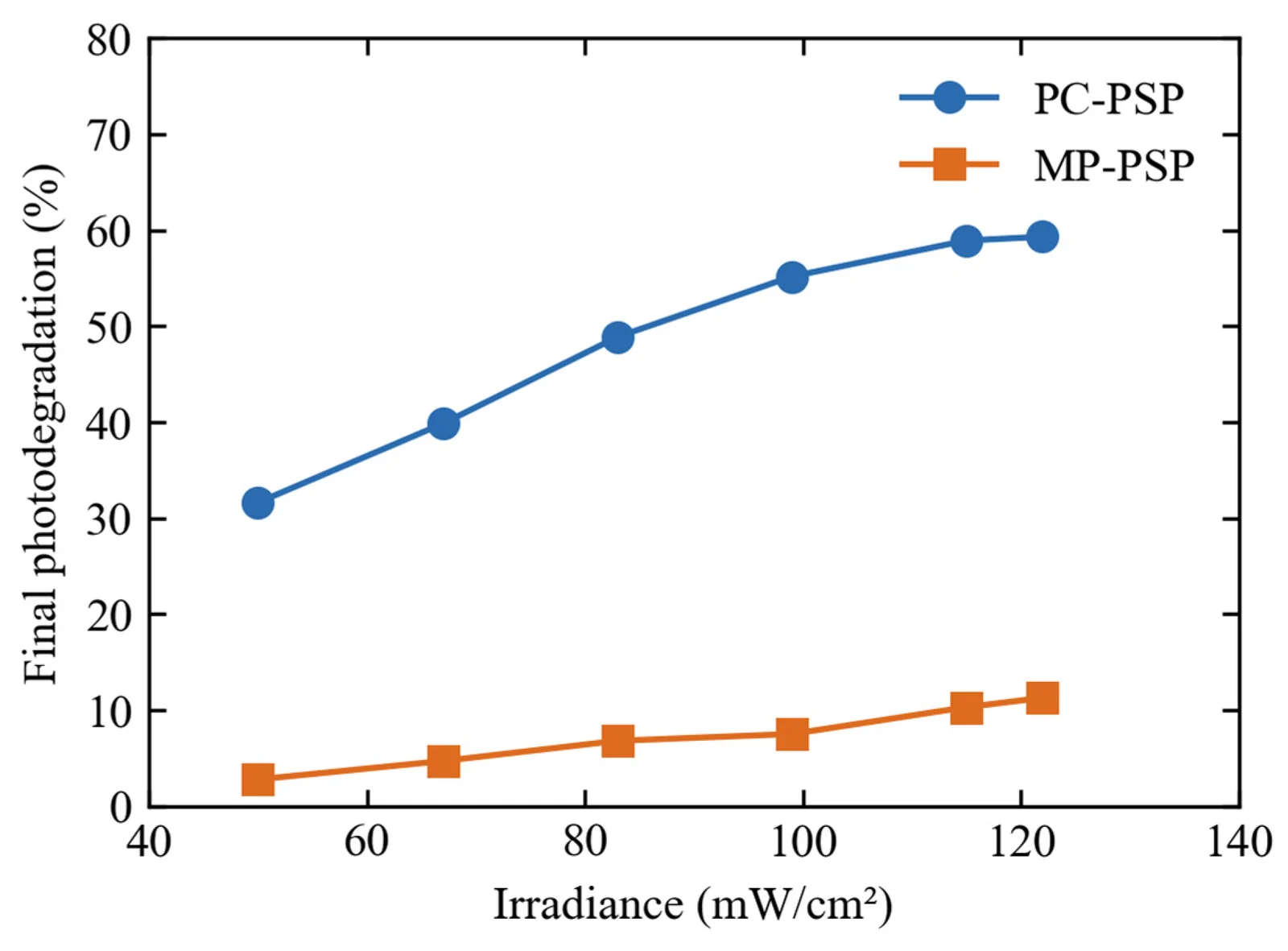
Final photodegradation ratios of PC-PSP and MP-PSP after 900 s of continuous irradiation as functions of irradiation intensity. The experiments were conducted at 100 kPa and 20 °C.

Figure 2a,b shows the normalized luminescence intensities of PC-PSP and MP-PSP under different irradiation intensities at 100 kPa. Both PSPs exhibited a relatively rapid initial decrease followed by a slower decay, and the bi-exponential model adequately described these temporal characteristics. PC-PSP exhibited substantially stronger photodegradation than MP-PSP. Figure 2c,d further presents the photodegradation ratio as a function of accumulated radiant exposure. The PC-PSP curves obtained at different irradiation intensities were relatively close, indicating that its degradation was largely determined by radiant exposure. In contrast, the MP-PSP curves remained separated at the same radiant exposure, demonstrating that equal radiant exposures obtained using different combinations of irradiation intensity and exposure time did not produce equivalent degradation. As shown in Figure 3, the final degradation ratio of PC-PSP increased from approximately 31% at 50 mW/cm^2^ to 49% at 83 mW/cm^2^ and reached approximately 59% at 115–122 mW/cm^2^. In comparison, that of MP-PSP increased gradually from approximately 3% to 11%. At 122 mW/cm^2^, MP-PSP retained approximately 89% of its initial intensity, whereas PC-PSP retained approximately 41%. The higher photostability of MP-PSP may be associated with the immobilization of luminophores within mesoporous silica particles, which reduces their direct interaction with the polymer and improves their spatial distribution [14]. In PC-PSP, the high local luminophore concentration and resulting dynamic self-quenching may accelerate photodegradation [15].

**Figure 4:**
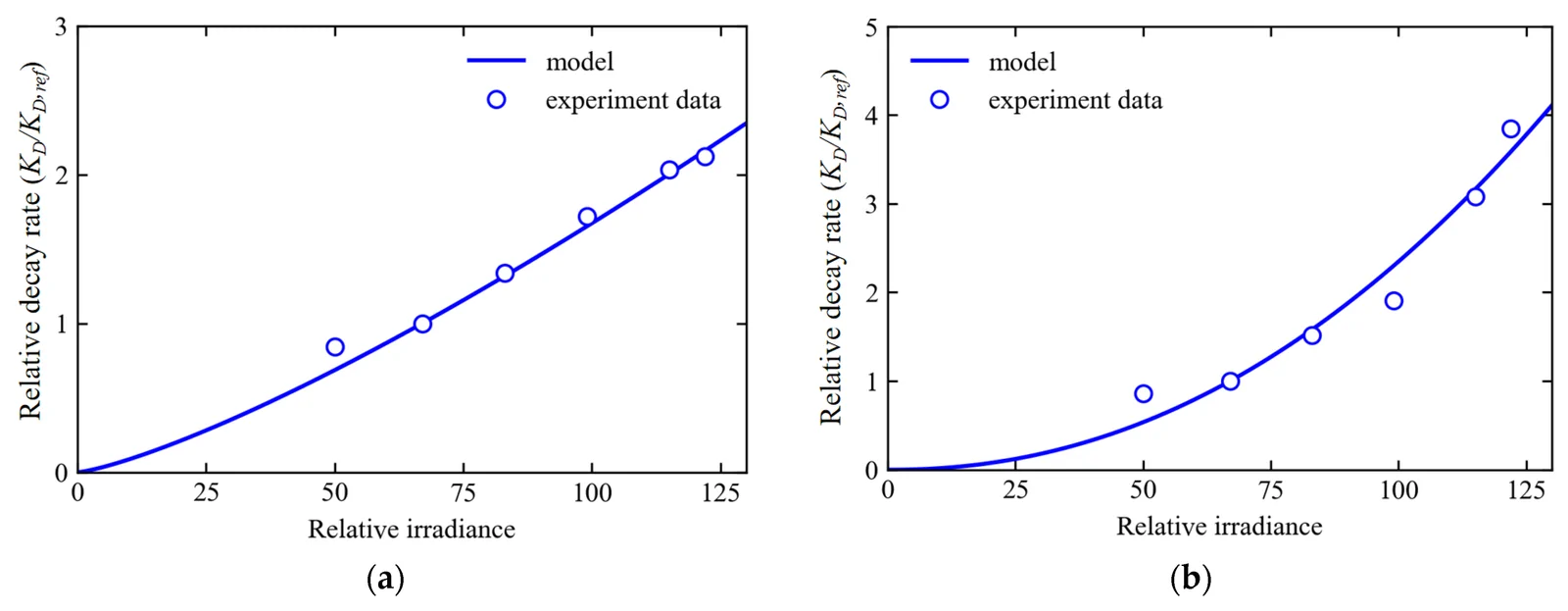
Relative composite degradation-rate constants as functions of irradiation intensity at 100 kPa: (**a**) PC-PSP; (**b**) MP-PSP. The degradation-rate constants were normalized by their respective values at 67 mW/cm^2^. Solid lines represent the power-law model, and symbols represent the experimental results.

Figure 4 presents the dependence of the relative degradation rate on irradiation intensity at 100 kPa. The power-law exponents were 1.28 ± 0.049 for PC-PSP and 2.13 ± 0.099 for MP-PSP. PC-PSP exhibited a near-linear dependence, with a twofold increase in irradiation intensity producing an approximately 2.43-fold increase in the degradation rate. In contrast, MP-PSP showed a much stronger nonlinear dependence, for which doubling the irradiation intensity increased the degradation rate by a factor of approximately 4.36. Although the absolute degradation of MP-PSP remained substantially lower, its degradation kinetics were more sensitive to irradiation intensity. The fitted exponents are consistent with the radiant-exposure behavior observed in Figure 2c,d. The near-linear irradiance dependence of PC-PSP corresponds to the relatively close radiant-exposure curves, whereas the larger exponent of MP-PSP explains the pronounced separation between its curves.

The near-unity exponent of PC-PSP at 100 kPa may indicates that its degradation rate remains relatively close to proportional to irradiation intensity, suggesting that oxygen-independent photodecomposition is likely the principal pathway, with only a limited contribution from nonlinear processes. For MP-PSP, m>2 may suggests a greater contribution from photooxidation. Increasing irradiation intensity may increase both the excited-state luminophore population and the formation of reactive intermediates (e.g., electronically excited oxygen molecules or polymer photodecomposition products), thereby potentially contributing to the strongly nonlinear increase in the degradation rate. These interpretations are inferred from the observed kinetic behavior, and further chemical or spectroscopic characterization is required to identify the underlying degradation pathways more precisely.

### 4.2. Effect of Pressure

**Figure 5:**
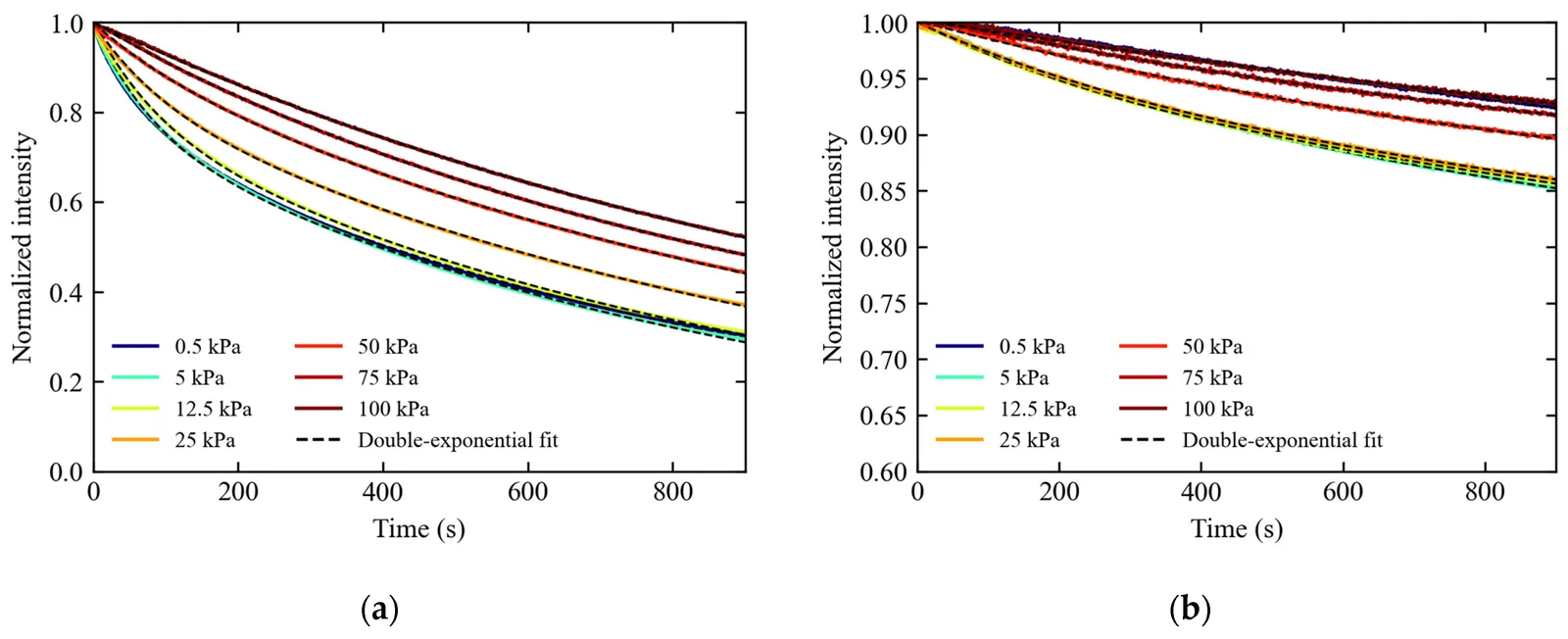
Normalized luminescence intensity histories of (**a**) PC-PSP and (**b**) MP-PSP at different pressures. The experiments were conducted for 900 s under continuous irradiation at 67 mW/cm^2^ and 20 °C. Dashed lines represent the bi-exponential fits.

**Figure 6:**
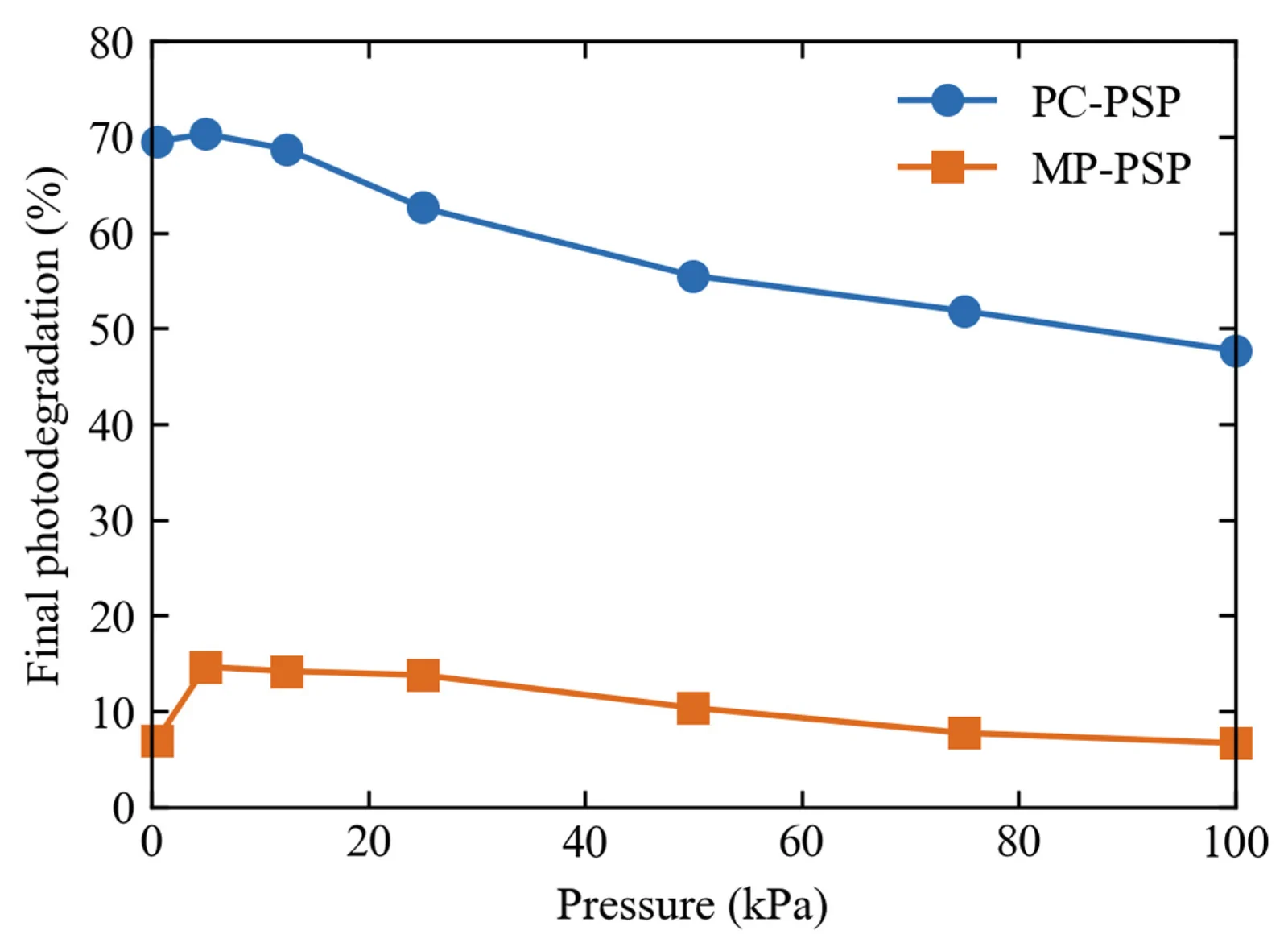
Final photodegradation of PC-PSP and MP-PSP after 900 s of continuous irradiation as a function of pressure. The experiments were conducted at 67 mW/cm^2^ and 20 °C.

Figure 5 and Figure 6 present the normalized intensity histories and endpoint photodegradation of PC-PSP and MP-PSP from 0.5 to 100 kPa. Both formulations exhibited continuous intensity loss, which was well reproduced by the bi-exponential model. From 5 to 100 kPa, increasing pressure reduced the final photodegradation from approximately 70% to 48% for PC-PSP and from 15% to 7% for MP-PSP. At 0.5 kPa, however, the final photodegradation was slightly lower than at 5 kPa for PC-PSP and decreased to approximately 7% for MP-PSP, indicating a reversal of the pressure dependence under oxygen-limited conditions. The overall decrease above 5 kPa is consistent with oxygen quenching, which reduces the excited-state luminophore population available for irreversible photochemical reactions, whereas the low-pressure reversal suggests suppression of oxygen-dependent photooxidation.

**Figure 7:**
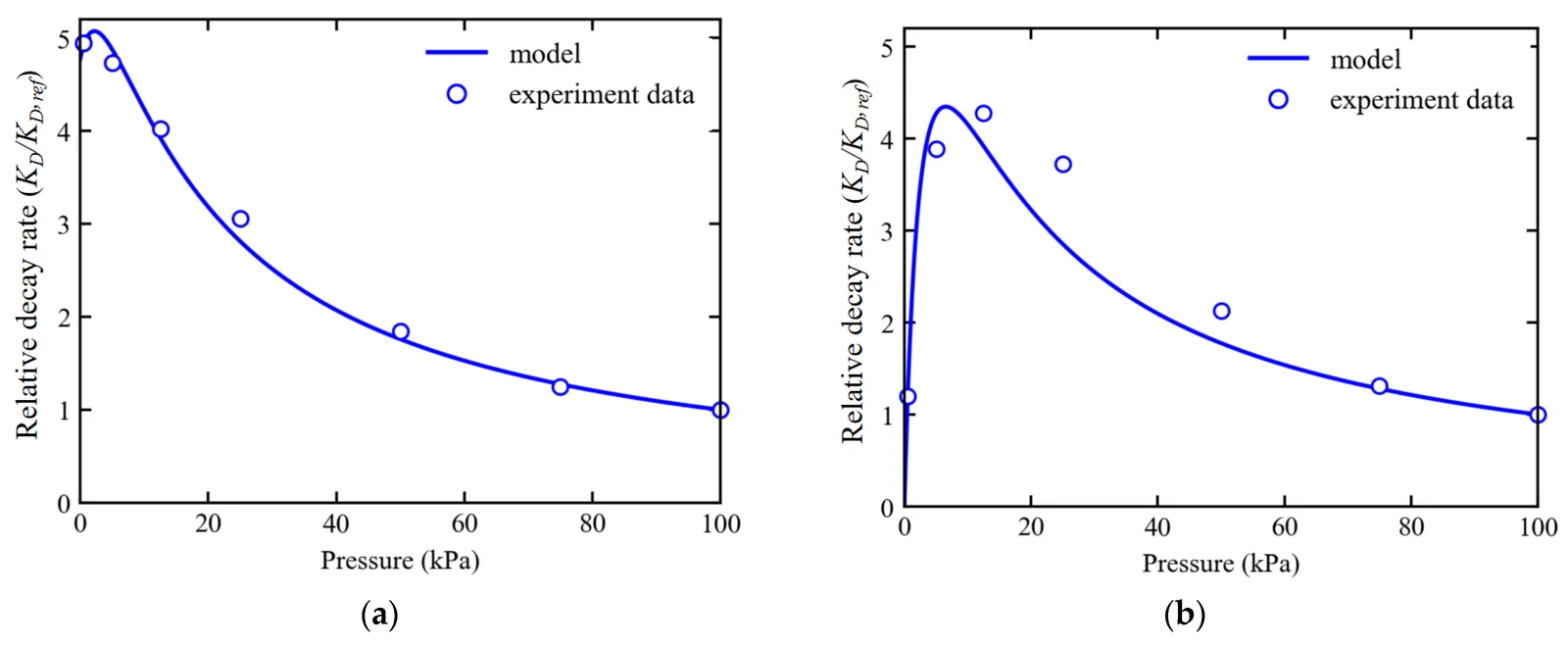
Relative photodegradation rates of (**a**) PC-PSP and (**b**) MP-PSP as functions of pressure. Symbols denote experimental values, and solid lines represent the half-saturation oxygen model fits obtained using Equation (8), with $P_{ref}=100$ kPa.

The relative degradation rates were fitted using the half-saturation oxygen model in Equation (8), as shown in Figure 7. For PC-PSP, the fit yielded $p_{O_{2},1/2}=1.92\pm 0.17$ kPa, $\beta_{ox}=1.64\pm 0.22$, and $K_{q}=0.52\pm 0.12$ kPa^−1^, with R^2^ = 0.9914 and RMSE = 0.13. The model reproduced the high relative degradation rate at low pressure and its gradual decrease toward the reference value at 100 kPa. For MP-PSP, the corresponding parameters were $p_{O_{2},1/2}=1.37\pm 0.37$ kPa, $\beta_{ox}=902\pm 82$, and $K_{q}=0.73\pm 0.16$ kPa^−1^, with R^2^ = 0.8759 and RMSE = 0.44. The fit captured the low-pressure maximum and the overall decrease at higher pressure, although some deviations remained at intermediate pressures.

For PC-PSP, the relatively small $\beta_{ox}$ and the nearly monotonic decrease in degradation rate indicate that oxygen quenching playing a major role in the pressure dependence of $K_{D}$ over most of the investigated range. In contrast, the much larger fitted $\beta_{ox}$ of MP-PSP suggests a stronger contribution from oxygen-dependent photooxidation. When the oxygen partial pressure falls below the half-saturation pressure, $p_{O_{2},1/2}=1.37$ kPa, corresponding to an air pressure of approximately 6.53 kPa, the degradation rate decreases rapidly with further pressure reduction. In this oxygen-limited region, the reduced oxygen concentration suppresses photooxidation, and this suppression outweighs the increase in degradation expected from weaker oxygen quenching. This behavior is consistent with the strongly nonlinear irradiance dependence observed in Section 4.1, which also suggests a greater photooxidative contribution to MP-PSP degradation. It should be noted that *β_ox_* is a dimensionless ratio of the oxygen-dependent photooxidation contribution to the oxygen-independent photodecomposition contribution. On one hand, a relatively small *K_pd_* may lead to a large *β_ox_*; on the other hand, the comparatively large fitting error for MP-PSP may increase the uncertainty in, and potentially amplify, this estimated ratio. Therefore, the value of *β_ox_* is not interpreted quantitatively but is used only, together with the low-pressure reversal and nonlinear irradiance dependence, as qualitative evidence of a substantial oxygen-dependent contribution to MP-PSP degradation. And these mechanistic assignments are based on the combined kinetic trends and do not constitute direct chemical identification of the degradation pathways.

To further examine the influence of pressure on the irradiance dependence of photodegradation, the power-law model in Equation (5) was applied to the irradiance-series results obtained at 100, 10, and 1 kPa. As summarized in Table 1, the model described the experimental trends well at all three pressures, with low uncertainty, indicating that the same general functional form remained applicable over the investigated pressure range. However, the fitted exponent *m* decreased systematically with decreasing pressure. For PC-PSP, m decreased from 1.28 at 100 kPa to 0.63 at 10 kPa and 0.52 at 1 kPa. For MP-PSP, it decreased from 2.13 to 1.26 and 1.08, respectively. The decrease in *m* is also consistent with the oxygen-limited behavior discussed above: as pressure and oxygen availability decrease, the relative contribution of photooxidation may be reduced, resulting in a weaker nonlinear dependence on irradiance. Taken together, the consistent applicability of the power-law model and the pressure-dependent variation in *m* provide preliminary insight into the coupling between pressure and irradiance, although a comprehensive characterization of this interaction requires further investigation.

### 4.3. Effect of Temperature

**Figure 8:**
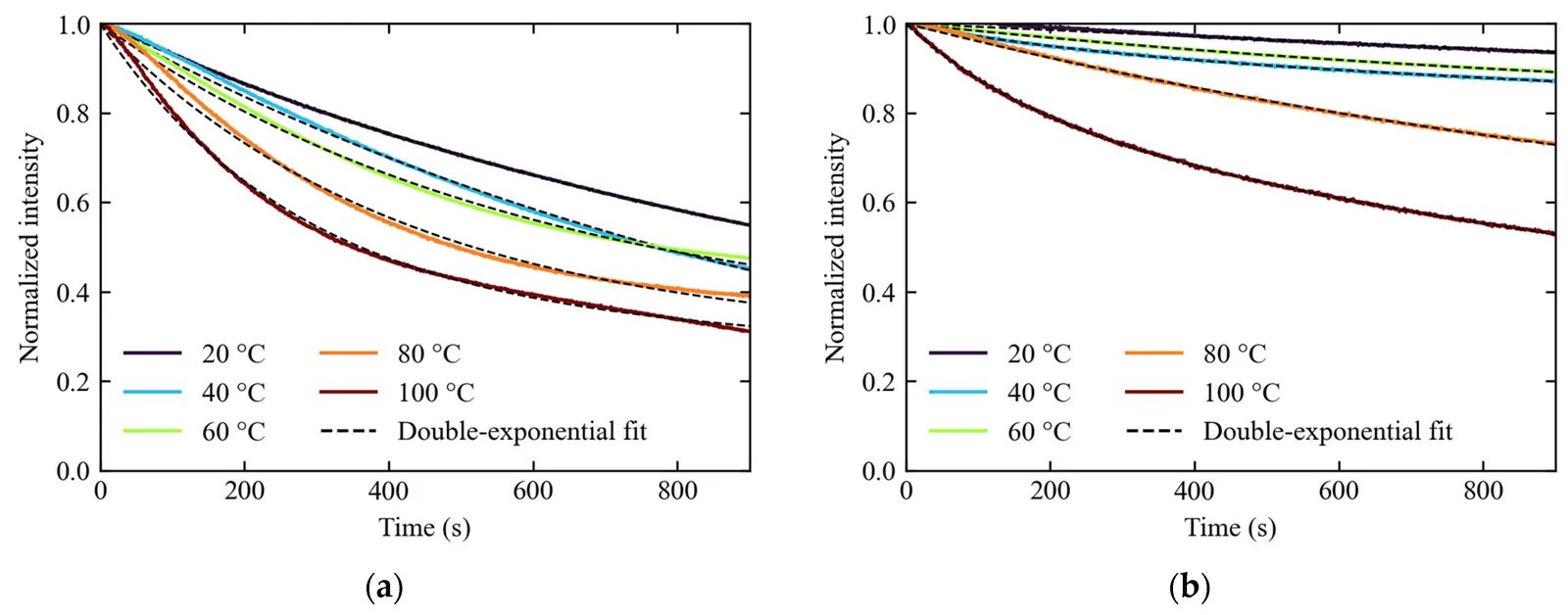
Normalized luminescence intensity histories of (**a**) PC-PSP and (**b**) MP-PSP at different temperatures. The experiments were conducted for 900 s at 67 mW/cm^2^ and 100 kPa. Dashed lines represent the bi-exponential fits.

**Figure 9:**
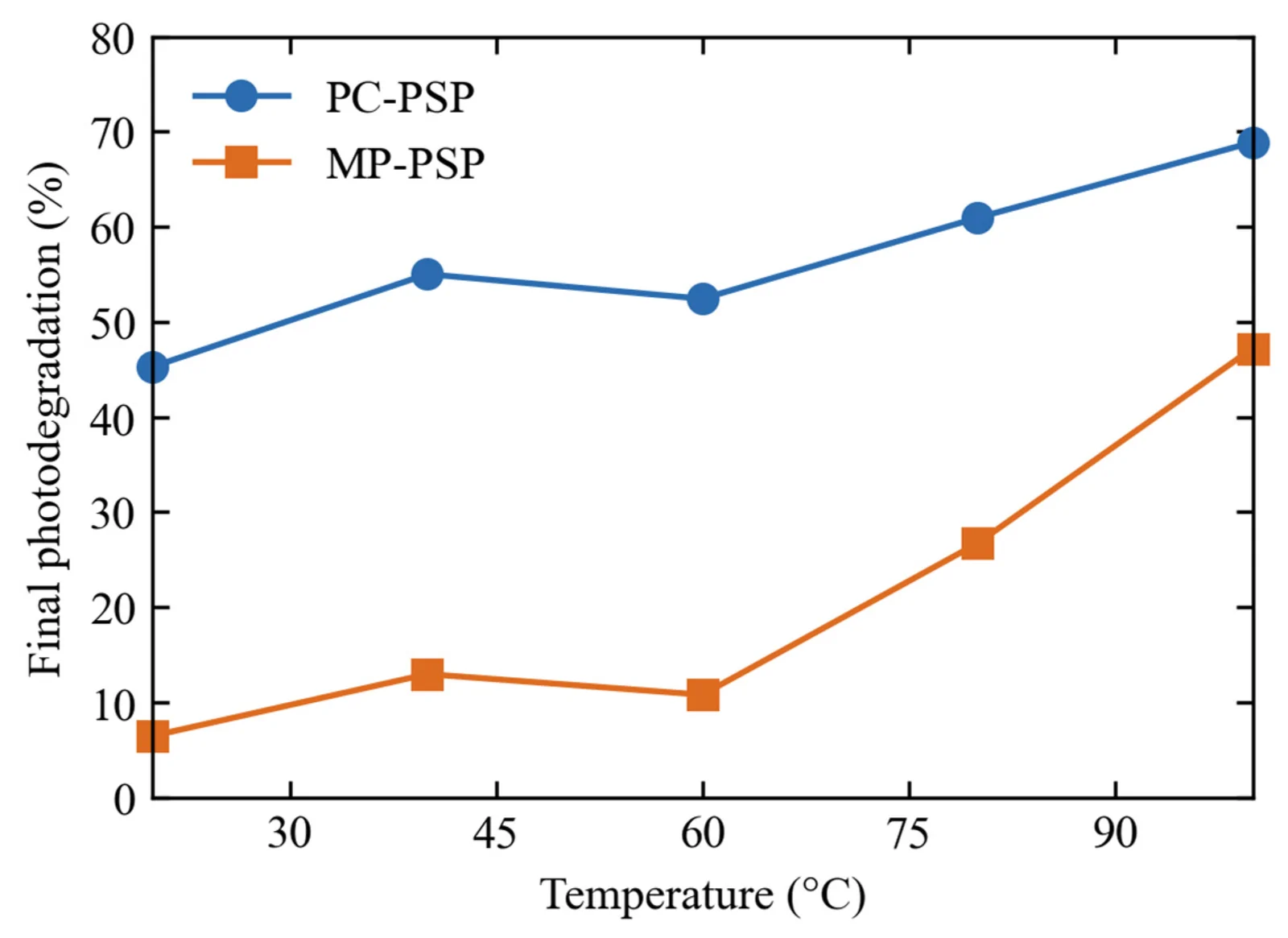
Final photodegradation of PC-PSP and MP-PSP after 900 s of continuous irradiation as a function of temperature at 67 mW/cm^2^ and 100 kPa.

Figure 8 shows an overall acceleration of photodegradation with increasing temperature. After 900 s, the normalized intensity of PC-PSP decreased from approximately 0.55 at 20 °C to 0.31 at 100 °C, whereas that of MP-PSP decreased from approximately 0.94 to 0.53. The bi-exponential model captured the principal decay trends, although the agreement was poorer than that obtained for the irradiance- and pressure-dependent results, particularly at elevated temperatures. These deviations suggest that additional degradation pathways or changes in the luminophore–binder microenvironment may contribute to more complex kinetics at high temperatures. Figure 9 further shows that the final photodegradation increased from approximately 45% to 69% for PC-PSP and from 6% to 47% for MP-PSP. PC-PSP exhibited a slight decrease between 40 and 60 °C, whereas the degradation of MP-PSP increased markedly above 60 °C, narrowing the difference between the formulations at 100 °C.

**Figure 10:**
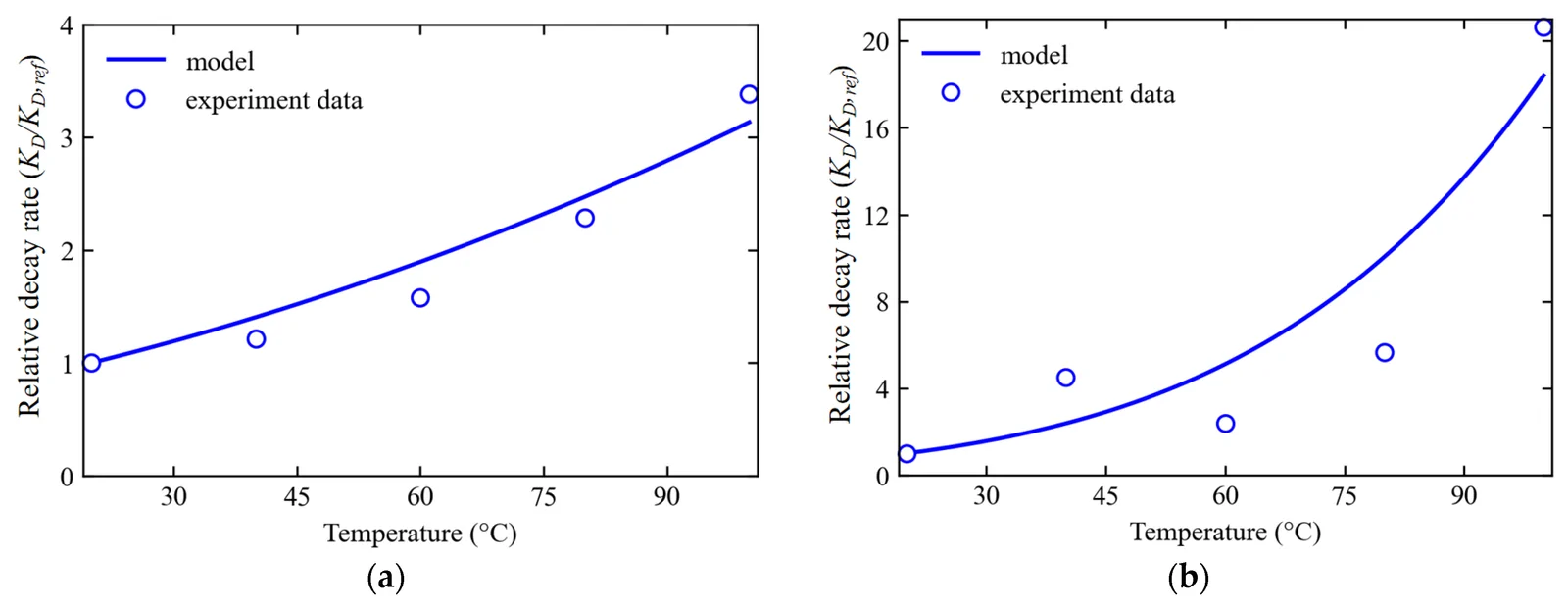
Relative photodegradation rates of (**a**) PC-PSP and (**b**) MP-PSP as functions of temperature. Symbols denote experimental values, and solid lines represent the Arrhenius fits obtained using Equation (10), with $T_{ref}=20$ °C.

Figure 10 compares the relative decay rates with the Arrhenius relation in Equation (10). An additional clarification is warranted regarding the physical interpretation of the fitted $E_{a}$. The Arrhenius-type model is intended to describe the overall temperature dependence of the composite degradation rate rather than the kinetics of an individual elementary reaction. For PC-PSP, the fitted activation energy was $E_{a}=12.99\pm 0.82\;{kJ\;mol}^{-1},$ with an RMSE of 0.241. The relatively low RMSE indicates that the increase in decay rate was adequately described by an Arrhenius-type temperature dependence. For MP-PSP, the fitted activation energy was $E_{a}=33.12\pm 1.95\;{kJ\;mol}^{-1},$ with an RMSE of 3.01. The higher activation energy indicates a stronger temperature dependence of the normalized decay rate. However, the experimental rates at 40–80 °C were non-monotonic and deviated appreciably from the fitted curve. The different fit qualities suggest that the observed temperature dependence may involve factors beyond the intrinsic rate of a single photochemical pathway.

It should be noted that temperature can also accelerate photochemical reactions, modify oxygen solubility and transport within the binder, and alter the polymer state and local luminophore environment, making its overall influence on photodegradation more complex than a single Arrhenius process. Because oxygen availability and the excited-state luminophore population are also affected by pressure and irradiance, their coupling with temperature may introduce additional complexity. The present results provide a preliminary characterization of the independent temperature dependence.

## 5. Conclusions

This study quantified the photodegradation of PC-PSP and MP-PSP under independently controlled irradiation intensity, pressure, and temperature. The nonlinear intensity histories were described using a bi-exponential function, and a composite initial degradation-rate constant was used to compare the two formulations. The main conclusions are as follows:

1. Increasing irradiation intensity accelerated photodegradation in both formulations, but their rate dependences differed. PC-PSP exhibited a near-linear dependence, with m=1.28, suggesting a greater relative contribution from oxygen-independent photodecomposition. MP-PSP showed a strongly nonlinear dependence, with m=2.13, which is consistent with a greater relative contribution from photooxidation.
2. Pressure affected photodegradation through the competition between oxygen quenching and oxygen-dependent photooxidation. For PC-PSP, the degradation rate decreased nearly monotonically with increasing pressure, which is consistent with oxygen quenching playing a major role in its pressure dependence over most of the investigated range. MP-PSP exhibited a non-monotonic response: the initial increase in degradation with decreasing pressure is consistent with weakened oxygen quenching, whereas the subsequent decrease below the half-saturation region suggests oxygen-limited suppression of photooxidation. Although the half-saturation model captured the overall pressure-dependent trend, deviations at intermediate pressures indicate that a single kinetic relation does not fully describe the behavior of MP-PSP. In addition, the irradiance exponent *m* decreased systematically with decreasing pressure for both PSPs, consistent with a reduced contribution of photooxidation as oxygen availability decreased.
3. Increasing temperature generally accelerated photodegradation in both formulations, with MP-PSP showing a stronger temperature dependence. From 20 to 100 °C, the final degradation increased from approximately 45% to 69% for PC-PSP and from 6% to 47% for MP-PSP. The deviations observed at elevated temperatures, particularly for MP-PSP, indicate that a single Arrhenius-type relation does not fully describe the temperature-dependent degradation behavior.

These findings provide a quantitative basis for assessing photodegradation-induced measurement errors, selecting appropriate operating conditions, and development of a comprehensive photodegradation-correction framework for time-resolved PSP measurements. The proposed pathway assignments are inferred from the combined kinetic trends and require direct chemical or spectroscopic measurements for further verification, and the coupled effects of irradiance, pressure, and temperature also require further investigation.

## Figures and Tables

**Table 1:** Irradiance exponents *m* of PC-PSP and MP-PSP at different pressures.

Pressure (kPa)	PC-PSP, m	MP-PSP, m
100	1.28 ± 0.049	2.13 ± 0.099
10	0.63 ± 0.021	1.26 ± 0.036
1	0.52 ± 0.033	1.08 ± 0.073

## Data Availability

The original contributions presented in this study are included in the article. Further inquiries can be directed to the corresponding author.

## Abbreviations and Nomenclature

Abbreviations	Definition
PSP	Pressure-sensitive paint
PC-PSP	Polymer–ceramic pressure-sensitive paint
MP-PSP	Mesoporous-particle pressure-sensitive paint
PtTFPP	Platinum(II) meso-tetra(pentafluorophenyl)porphine
poly(IBM)	Poly(isobutyl methacrylate)
LED	Light-emitting diode
UV	Ultraviolet
RMSE	Root-mean-square error
Symbol	Definition
*D*(*t*_e_)	Accumulated photodegradation ratio after an irradiation period (t_e)
*E*_irr_	Irradiance
*E*_irr,ref_	Reference irradiance
*E*_a_	Apparent activation energy
*f*	Initial contribution of the fast-decaying component
*I*(*t*)	Luminescence intensity at irradiation time (t)
*I*_0_	Luminescence intensity at the beginning of irradiation
*k*_1_	Fast degradation-rate constant
*k*_2_	Slow degradation-rate constant
*K*_D_	Composite degradation-rate constant
*K*_D,ref_	Composite degradation-rate constant at the reference condition
*K*_ox_	Limiting oxygen-dependent photooxidation contribution
*K*_pd_	Oxygen-independent photodecomposition contribution
*K*_q_	Effective oxygen-quenching coefficient
*m*	Irradiance power-law exponent
*p*	Total absolute pressure
*p*_ref_	Reference total pressure
*p*_O_2__	Oxygen partial pressure
*p*_O_2_,ref_	Reference oxygen partial pressure
*p*_O_2_,1/2_	Effective oxygen half-saturation pressure
*R*(*t*)	Normalized luminescence intensity
*R*_g_	Universal gas constant
*R* ^2^	Coefficient of determination
*t*	Irradiation time
*t*_e_	Irradiation duration used to evaluate accumulated degradation
*T*	Absolute temperature
*T*_ref_	Reference absolute temperature
*x*_O_2__	Oxygen mole fraction
*β*_ox_	Ratio of the limiting photooxidation contribution to the photodecomposition contribution, *K*_ox_/*K*_pd_
*θ*_O_2__	Relative oxygen concentration term for photooxidation
